# Potentially harmful elements pollute soil and vegetation around the Atrevida mine (Tarragona, NE Spain)

**DOI:** 10.1007/s10653-023-01591-y

**Published:** 2023-05-20

**Authors:** L. Roca-Perez, R. Boluda, J. A. Rodríguez-Martín, J. Ramos-Miras, P. Tume, N. Roca, J. Bech

**Affiliations:** 1 Dept. Biologia Vegetal, Facultat de Farmàcia, Universitat de València, Av. Vicent Andrés I Estellés s/n, 46100 Burjassot, Valencia Spain; 2 Department of Environment, Instituto Nacional de Investigación y Tecnología Agraria y Alimentaria (INIA), ES, 28040 Madrid, Spain; 3 Departamento de Didácticas específicas, Facultad de Ciencias de la Educación, Campus Universitario Menéndez Pidal, Avda. San Alberto Magno s/n, 14071 Córdoba, Spain; 4 Facultad de Ingeniería, Universidad Católica de la Santísima Concepción, Casilla 297, Concepción, Chile; 5 Departament de Biologia Evolutiva, Ecologia i Ciències Ambientals, Fac. Biologia, Universitat de Barcelona, Av. Diagonal 643, 08023 Barcelona, Spain; 6 Universitat de Barcelona (UB), Gran Via de les Corts Catalanes, 585, 08007 Barcelona, Spain

**Keywords:** Heavy metals, Plant, Soil, Barite mine

## Abstract

Mining activity is one of the main sources to pollute soil, water and plants. An analysis of soil and plant samples around the Atrevida mining area in Catalonia (NE Spain) was preformed to determine potentially harmful elements (PHEs). Soil and plant samples were taken at eight locations around the mining area. The topsoil (0–15 cm) samples were analysed for physico-chemical properties by standard methods, by ICP-MS for Cd, Co, Cr, Cu, Fe, Ni, Pb and Zn, and were microwave-digested. Plant, root and shoot samples were digested separately, and heavy metals were analysed by AAS. Translocation factor (TF), biological concentration factor (BCF) and biological accumulation factor (BAF) were determined to assess the tolerance strategies developed by native species and to evaluate their potential for phytoremediation purposes. Soil pH was generally acid (5.48–6.72), with high soil organic matter (SOM) content and a sandy loamy or loamy texture. According to the agricultural soil values in southern Europe, our PHEs concentrations exceeded the toxicity thresholds. The highest root content of the most studied PHEs appeared in *Thymus vulgaris* L. and *Festuca ovina* L., while *Biscutella laevigata* L. accumulated more PHEs in shoots. The TF values were > 1 in *B. laevigata* L., but BAF obtained < 1, except Pb. *B. laevigata* L., and can be considered potentially useful for phytoremediation for having the capacity to restrict the accumulation of large PHEs amounts in roots and Pb translocation to shoots.

## Introduction

Of all anthropogenic activities, mining is considered to contribute significantly to environmental pollution (Azevedo-Silva et al., 2016; Martinez-Carlos et al., 2021; Reyes et al., 2020). Either active or abandoned mining activities are a source of pollution by potentially harmful elements (PHEs) in the environment, particularity in soil, water and plants (Bech et al., 2012a, 2012b, 2016; Ko et al., 2020; Martinez-Carlos et al., 2021; Nguyen et al., 2020; Reyes et al., 2020). Deposits and veins of barite (Ba) and mining activity associated with Ba extraction can generate PHEs in soil, water and plants (Alizadeh-Kouskuie et al., 2020). Ba deposits are usually associated with sulphide minerals (Lottermoser, 2010), which can increase environmental PHEs pollution. One example is ore deposits from the old fluorite and Ba mines located in Hammam Zriba in Northern Tunisia for being main sources of particulate pollutants, which are continuously emitted and deposited at several distances with consequent soil pollution (Djebbi et al., 2017). Adamu et al (2015) described pond and stream pollution by Fe, Hg and Pb in the vicinity of a mine used to extract Ba. Mining activity performed to extract Ba has been associated with bigger amounts of Cd in soil used for rice cultivation near mining areas (Lu et al., 2019), and higher Ba, Pb and Sn contents in soils have been observed around a Ba mine in SE Nigeria (Ochelebe et al., 2020). A study performed around Las Herrerias mine (Almería, SE Spain) noted that Ba-rich ore stockpiles and mining waste dumps exposed to weathering processes can lead to the mobilisation of Ag, Al, Ba, Cu, Cd, Eu, Fe, Mn, Ni, Sb, Pb and Zn. Mine waste from this area shows high concentrations of Ag, As, Ba, Fe, Hg, Sb, Eu, Pb, Zn and Mn, and high soil concentrations of Ag, Ba, Fe, Sb, Pb, Zn, Mn, Cd and Eu (Navarro & Cardellach, 2009).

Wild plant species growing in the vicinity of mining areas with high PHEs contents in soil have been studied by different researchers from several countries (Bech et al., 2012a, 2012b, 2016; Wu et al., 2021) because it allows ecotypes to be identified with accumulative capacity for heavy metals, and metalloids to be used in phytoremediation. The factors that influence solubility and form available PHEs species in soil widely vary geographically, and include the concentration and chemical form of the elements entering soil, soil properties and soil processes because they influence the kinetics of sorption reactions, metal concentrations in solution, and the form of soluble and insoluble chemical species (Cataldo and Wildung, 1978). Different indices appear in the literature to determine heavy metal accumulation. Root (RAF) and shoot accumulation factors (SAF) describe the capability of roots and shoots to, respectively, accumulate PHEs from soil (Bech et al., 2012b, 2016; Ortiz-Oliveros, et al., 2021). The RAF and SAF indices are equivalent to biological concentration factor (BAF) and biological accumulation factor (BAF), respectively, as they are used to determine the capacity of plants to accumulate and transfer from soils to roots and shoots according to different authors (Hosseini et al., 2022; Korzeniowska & Stanislawska‑Glubiak, 2019). Hyperaccumulators are characterised by: (a) the shoot to root metal concentration ratio (i.e. translocation factor, TF) being over 1 (Subpiramaniyam, 2021); (b) the shoot to soil metal concentration ratio (i.e. SAF) exceeding 1 (Vysloužilová et al., 2003). Plants can be considered hyperaccumulators when SAF and TF are higher than 1 (Bech et al., 2012a, 2012b, 2016; Buscaroli, 2017) and suitable for phytoextraction (Hosseini et al., 2022; Tavili et al., 2021).

The identification and use of hyperaccumulator plants in mining projects have been acknowledged as an important component part of mine planning at several sites worldwide (Erskine et al., 2018). *Plantago orbignyana* L. (Bech et al., 2012b) *Senecio* sp. (Bech et al., 2012a), *Cortaderia hapalotricha* (Pilg.) Conert (Bech et al., 2016) and *Boehmeria nivea* (L.) Gaudich (Wu et al., 2021) with TF values above 1 are good examples of accumulator plants for specific heavy metals. Arsenic phytoextraction of mine waste using *Pteris vittata* L. has been developed and tested in China, Australia, the UK and the USA (Corzo Remigio et al., 2020). Two Cd hyperaccumulator examples are *Noccaea Caerulescens,* which is distributed in metalliferous and non-metalliferous soils in W Europe (Martos et al., 2016), and *Sedum plumbizincicola*, discovered close to a Pb–Zn mining area in China (Wu et al., 2013). Of the 69 species studied at the Tang-e Douzan mine in Isfahan (Iran), only *Cerastium dichotomum* and *Muscarineglectum* for Pb, *Ceratocephala falcata*, *M. neglectum*, *Ornithogalum orthophyllum* and *Ranunculus arvensis* for Zn and *C. falcata*, *M. neglectum*, *O. orthophyllum* and *R. hybrida* for Cd, have been suggested as being the most effective species for the phytostabilisation of polluted soils (Hesami et al., 2018). The existence of potentially toxic elements in hyperaccumulator plants in abandoned mines offers a unique opportunity for remediation by applying phytoextraction (van der Ent et al., 2012), and must be more widely explored.

Very few phytoremediation studies have been carried out in areas affected by Ba mining. Species like *Cassia angustifolia* and *Tephrosia purpurea* are able to bioaccumulate Sr and Zn in pollution areas around a Ba mine in India (Raghu, 2001). In Pb and Ba mines in the Iberian Peninsula, *Silene sclerocarpa* accumulates more heavy metals than *Simlax aspera* (Poschenrieder et al., 2012).

Currently, no study has analysed the content of potentially toxic elements in the soils and plants surround the Atrevida barite mine (NE Spain), and very few studies have been published in the Iberian Peninsula (Poschenrieder et al., 2012) for this specific mining activity type. The aim of this paper was to determine some PHEs concentration in the soil, roots and shoots of the plant species growing around the old Ba Atrevida mine (Tarragona, NE Spain) to establish the pollution levels of soil and plants, and to evaluate if the plant species from the soils close to this mine can be useful in phytoremediation.

## Material and methods

### Site description and sampling

**Fig. 1:**
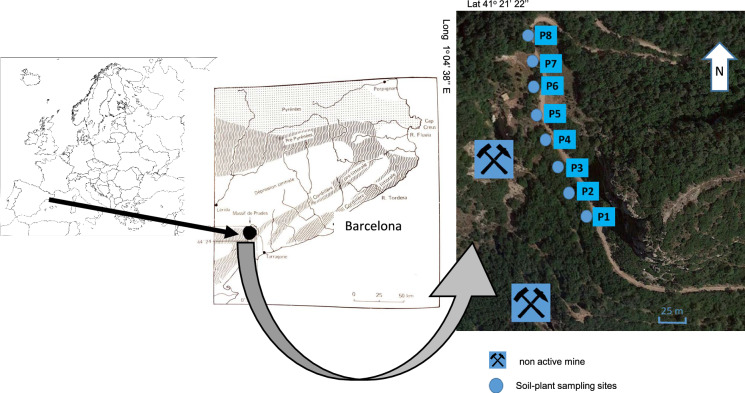
Soil and plant sampling site next to the Atrevida mine

The study area (Fig. 1) is located near the peak l’Aliga (1052 m) that lies south of the Poblet Monastery in the Vimbodí-Poblet municipality on the northern border of the Prades Mountains, SW of the Catalonian Coastal Ranges to NW of Tarragona (NE Spain). The Atrevida mine is located at latitude 41° 21′ 23′′ north, longitude 1º 4′ 45′′ east and at an altitude of 986 m (IGME, 1973).

The Atrevida mine exploitation peaked in the nineteenth and mid-twentieth centuries, and was still operating until the second half of the twentieth century. There are indications that the exploitation of the mine began in the middle ages. It had an aerial lift to transport minerals from the mine to trucks on the Poblet-Prades road, approximately 1 km from the Poblet Monastery. The mine was closed in 1990. Atrevida mineralisation consists of a powerful subvertical vein that is more than 3 km long in a NNW–SSE direction, 1–6 m thick and 150 m deep. In stratigraphic terms, this ore crosscuts black shale and quartzite of Upper Ordovician, black shales of Wenlock and Ludlow Series of Silurian, and conglomerates of facies Culm of the Carboniferous age. These Palaeozoic series are affected by the Hercynian orogeny producing NW–SE trending folds and thrusts and granite intrusion. This granite ranges in composition from quartz diorites to leucogranites rich in biotite and oligoclase (Bech et al., 1980), and produces very low-grade contact metamorphism. The Paleozoic series are discordant, overlain by conglomerates, sandstones and lutites of Buntsandstein facies. The alpine orogeny affects the aforementioned materials by producing NW–SE trending faults, which were reactivated during the Neogene and contain mineralised strati-form massive sulphides and veins of banded and brecciated Ba, locally cemented by a complex polymetallic assemblage of sulphides, arsenides and sulpharsenides. In the Atrevida mine, numerous minerals like Ba, fluorite, quartz, chalcopirite, galena, sphalerite, azurite, malaquite, marcassite, annabergite, arsenolite, maucherite, pyrite, millerite, nickeline, pyromorphite, calcantine and native silver, and many more, have been found (Mata-Perelló, 2011; Melgarejo & Ayora, 1985; Viñals et al., 1990).

The climate type is submediterranean (Köpen climate type is Csa). The mean annual temperature is 11 °C, with a minimum of 4 °C from December to February, and a maximum of 19.6 °C between July and August. The mean annual rainfall is 800 mm with three maximums in February, May and November. The predominant vegetation in the area is the *Quercetum mediterraneo-montanum* forest comprising shrubs and trees with *Quercus pubescens* Wild*, Q. ilex* L.*, Q. valentina* Cav.*, Q. pyrenaica* Wild*, Castanea sativa* Mill.*, Pinus sylvestris* L.*, P. pinaster* Aiton*, P. nigra* Arnold*, Arbutus unedo* L.*, Cistus laurifolius* L.*, C. albidus* L. and *Arctostaphylos uva ursi,* var. *crassifolia* (L.) Spreng. The soils in the study area present a mesic soil temperature regime and a xeric soil moisture regime (Bech et al., 1983), classified as Xeralfs (Soil Taxonomy, 2014) and Luvisols (IUSS Working Group WRB, 2015).

Soil sampling was carried out at eight sites (P1, P2, P3, P4, P5, P6, P7 and P8) located around the abandoned mine (Fig. 1). Systematic and focused sampling was carried out. The sampling area corresponds to a high plateau of gently undulating topography with small watercourses, where sites P2, P3, P4 and P8 are higher, and P1, P5, P6 and P7 are closer to the watercourse bed. The commonest plant species were collected in the zone. Surface soil samples (15 cm depth), except for sample P2 that was an organic horizon, were taken at approximately 20-m intervals. A variable number of plant species was collected at each site, with 23 species in all.

### Soil and plant analysis

All the soil samples were air-dried, sieved with a 2-mm grid and stored in hermetically sealed polyethylene bags until analysed. Soil pH was measured in soil:water suspension 1:5 v/v (UNE, 1999), and electrical conductivity (EC) was measured by suspension of soil in water at a ratio of 1:5 w/v (UNE, 2001). Soil organic matter (SOM) was determined by the Walkley–Black method (Walkley & Black, 1934). Total nitrogen, cation exchange capacity (CEC) and particle size distribution were carried out following the analytical methods described by Roca-Perez et al. (2002).

The Cd, Co, Cr, Cu, Fe, Ni, Pb and Zn contents in soil and plants were determined. For this purpose, approximately 0.5–1 g of the dried ground sample (particle size < 150 μm) was acid-digested with a mixture of 4 ml of concentrated nitric acid, 30 ml of concentrated hydrogen peroxide (33%), 2 ml of sulphuric acid and 5 ml of deionised water (EPA, 2007, modified) in a microwave oven (Mars, CEM Corp, Matthews, NC, USA) at 220 °C for 30 min. Plant material was washed with distilled water, divided into shoots and roots, and then dried at 60 °C and pulverised. Between 0.5and 1 g of sample was digested in a microwave oven at 200 °C for 20 min using a mixture of nitric acid (65%) and hydrogen peroxide (30%) at a ratio of 5:1, respectively (Bech et al., 2012a). Three replicates were performed per sample. The concentrations of the elements in the soil and plant digestions were determined by ICP-OS (THERMO ICAP 6500DUO) and AAS (AAS-Thermo Solaar S2-2006), respectively. The detection limits of elements in mg L^−1^ for ICP-OS were 0.001 for Cd, Co, Cr, Ni, Cu and Pb, and 0.014 for Fe and Zn; on the other hand, limit of quantification of all elements for ICP-OS was found between 0.53 mg kg^−1^ for Cd, Co, Cr, Ni, Cu and Pb, and 5.96 mg kg^−1^ for Fe and Zn. Analysis quality control was carried out by using certified soil material (Silt Loam 1 trace Metals, CRM044-050, RTC) and plant reference material (Citrus leaves, NCS ZC 73,018). The percentage recoveries of the elements in the certified sample are shown in Table 1.

**Table 1:** Recovery percentage for each element analysed by ICP-OS in certified soil material and by AAS in certificated plant material

Element	Recovery (%)			
	Silt loam soil		Citrus leaves	
	Mean	SD	Mean	SD
Cd	90.10	5.87	119.41	9.78
Co	80.26	7.02	149.12	15.02
Cr	92.39	8.86	100.99	9.55
Cu	81.79	7.14	90.59	0.50
Fe	85.84	7.83	102.97	0.36
Ni	86.77	7.63	119.75	9.62
Pb	70.24	6.80	115.69	2.53
Zn	83.37	6.65	90.66	1.05

Mean and standard deviation (SD), *n* = 3

### Calculation PHEs indices

The BCF = [C_R_]/[Cs], where C_R_ and Cs indicate the element concentration (mg kg^−1^) in plant roots and soil, respectively. The BAF = [*C*_*A*_]/[*Cs*], where *C*_*A*_ and *Cs* show the element concentration (mg kg^−1^) in plant shoots and soil, respectively (Korzeniowska & Stanislawska‑Glubiak, 2019; Hosseini et al., 2022). Finally, the TF = [*C*_*A*_]/[*C*_*R*_], where *C*_*A*_ and *C*_*R*_ indicate the element content (mg kg^−1^) in plant shoots and roots, respectively (Bech et al., 2012b; Hosseini et al., 2022; Subpiramaniyam, 2021).

### Statistical analysis

All the analysed variables were firstly tested for data normality by the Shapiro–Wilk test. The Pearson or Spearman correlation analysis was done to relate the concentrations among not only the various elements and also to soil properties, but also among the elements in soil and different plant parts. All the analyses were performed by the IBM Statistics SPSS 26 package.

## Results and discussion

### Soil properties

Table 2 offers the general soil sample characteristics. Clay content varied from 8 to 21%. Soil texture was loamy (P1, P3 and P6) and loamy sandy (P4, P5, P7 and P8). Soil pH (5.48–6.72) was slightly and moderately acid. All the samples presented low EC, except site P2 (0.599 dS m^−1^) with a high and very high SOM content because its sampling site lay in the forest area. CEC was moderate and high as regards textural class. Soil P2 obtained the highest EC, Nt and CEC values due to a very high SOM content. These results generally suggest that the cation retention in these soils had to be high, and bioavailability could be considerable due to the acidic pH.

**Table 2:** Soil characteristics (mean ± standard deviation; *n* = 3) around the abandoned barite mine from Poblet (Tarragona, Spain)

Site	Sand (%)	Silt (%)	Clay (%)	pH	EC (dS m^−1^)	SOM (%)	Nt (%)	CEC (cmol_c_ kg^−1^)
P1	45 ± 2	38 ± 1	17 ± 2	6.54 ± 0.03	0.224 ± 0.009	11.82 ± 0.51	0.47 ± 0.03	18.85 ± 1.37
P2*	na	na	na	6.66 ± 0.01	0.599 ± 0.009	48.29 ± 3.62	1.24 ± 0.07	44.23 ± 8.81
P3	44 ± 1	35 ± 6	21 ± 5	6.67 ± 0.02	0.150 ± 0.002	4.92 ± 0.28	0.20 ± 0.01	12.95 ± 1.46
P4	60 ± 4	29 ± 1	11 ± 3	6.72 ± 0.04	0.148 ± 0.010	5.62 ± 0.02	0.29 ± 0.01	13.62 ± 0.79
P5	71 ± 5	20 ± 3	9 ± 1	6.17 ± 0.05	0.172 ± 0.004	16.49 ± 0.41	0.66 ± 0.04	21.79 ± 2.30
P6	42 ± 3	38 ± 2	20 ± 3	6.62 ± 0.04	0.143 ± 0.006	8.43 ± 0.33	0.42 ± 0.02	16.70 ± 3.04
P7	66 ± 7	26 ± 1	8 ± 1	5.48 ± 0.05	0.129 ± 0.009	6.43 ± 0.53	0.32 ± 0.03	12.92 ± 3.68
P8	51 ± 6	39 ± 2	10 ± 1	6.01 ± 0.02	0.109 ± 0.004	14.27 ± 0.27	0.51 ± 0.01	23.86 ± 2.70

*EC* electrical conductivity, *SOM* soil organic matter, *Nt* total nitrogen, *CEC* cation exchange capacity and *na* not analysed

*Organic horizon

### Soil PHEs concentration

The PHEs content in the soil the near the Atrevida mine are shown in Table 3. The values for each element indicate wide variation between certain sampling sites despite the relative proximity among them. The mean concentrations of the elements in the Atrevida mine soils followed this descending order: Fe > Zn > Pb > Cu > Cr > Ni > Co > Cd. The highest PHE contents were particularly noted at P1. High values were found at P5, P6 and P7, and the lowest values at P2 and P4. This result indicates a heterogeneous distribution in the content of these elements among the different sampling sites. This finding could be related to the fact that materials were dragged from the mine by runoff waters, and as indicated in material and methods, the sampling sites at higher elevations (P2, P4 and P8) show a lower concentration of these elements compared to the lower elevations sites (P1, P5, P6 and P7) which accumulate particles from the mine and therefore increase the content of PHEs in these sites. In addition, the differences in organic matter and clay contents could help to increase the differences among the studied sites.

**Table 3:** Concentration (mg kg^−1^) of PHEs in the different soils sites around the abandoned barite mine from Vimbodí-Poblet (Tarragona, NE Spain)

PHEs		Sample site								Mean of all sites
		P1	P2	P3	P4	P5	P6	P7	P8	
Cd	Mean	7.37	2.13	2.18	1.69	5.93	2.50	2.64	3.04	3.44
	SD^a^	0.54	0.14	0.18	0.11	0.36	0.16	0.18	0.21	
Co	Mean	130.59	14.78	59.62	27.97	177.12	56.10	40.18	42.72	68.63
	SD	9.91	0.89	4.17	2.40	12.12	3.44	3.01	3.56	
Cr	Mean	65.83	22.71	50.21	35.88	66.60	62.59	388.28	80.39	96.56
	SD	4.83	2.14	3.01	2.61	3.40	4.49	29.41	4.46	
Cu	Mean	357.42	26.43	114.00	26.11	119.87	55.53	309.93	325.31	166.82
	SD	34.98	2.06	10.93	2.25	9.99	3.43	26.74	25.61	
Fe	Mean	121,620	16,684	38,692	38,646	101,072	48,109	139,340	63,808	70,996
	SD	6767	1300	2547	2739	6032	2924	8294	3488	
Ni	Mean	156.67	16.85	75.08	28.85	138.48	111.36	85.65	122.01	91.87
	SD	8.43	0.84	4.73	1.73	7.23	7.46	4.34	9.64	
Pb	Mean	1146.14	49.25	158.21	166.77	1162.10	163.39	171.03	221.66	404.94
	SD	112.66	4.26	10.92	12.05	69.73	11.86	16.84	20.19	
Zn	Mean	2232.59	207.19	388.80	439.62	1268.40	481.90	282.16	475.04	721.96
	SD	120.11	12.43	25.27	31.65	71.03	28.91	14.39	23.75	

^a^Standard deviation

Several studies indicate that PHEs tend to accumulate in soil affected by mining activities. The mean Cu, Pb and Zn values were lower than the values obtained in most of the soils surrounding a polymetallic Caroline mine in the Peruvian Andes (Bech et al., 2016), and the Cu values reported in the soils around mining waste deposits to the NE of Taltal city (Chile) (Reyes et al., 2020). However, the mean Cu, Pb and Zn values obtained in this study were higher than those found in the vicinity of a tungsten open-pit Nui Phao mine in Vietnam (Nguyen et al., 2020). A comparison of the element soil concentrations in the vicinity of the Atrevida mine to other deposits, veins or Ba mines from other countries revealed that the mean values for the Cd, Co, Cr, Cu Ni, Pb and Zn concentrations at the Atrevida mine were higher than that those of deposits from the Mangampeta and Vemula Ba mining areas in India (Raghu, 2001). Nevertheless, Cd, Co and Cr were similar, Cu and Ni were lower, and Pb and Zn were higher than the Ba soils in Iran (Alizadeh-Kouskuie et al., 2020). The Cd values fell within the range reported by previous research works in soils near the Dahebian Ba mine in China (Lu et al., 2019), and the mean Co soil value was higher than those reported in soils around the Las Herrerias mining district (Almería, Spain) (Navarro & Cardellach, 2009) and at a fluorite mine in NE Tunisia (Djebbi et al., 2017).

In the present study, most of the concentrations of the studied PHEs (Cd, Co, Cr, Cu, Ni and Pb) at many sites were higher than the median values for agricultural soil (0–20 cm) in S Europe; Co at P1 and P5, and Zn exceeded the maximum value found in those agricultural soils (Reimann et al., 2018). It should be noted that the mean Cd, Co, Cr, Cu, Ni, Pb and Zn concentrations generally fell within the range of the values reported by the soil guideline value (SGV) for the EU (Reimann et al., 2018). However, Co and Pb at P1 exceeded the maximum SGV range value when the contents at each sampling site were individually analysed.

Compared to Spanish soils, the Cd, Co (except P1, P4 and P5) and Zn (except P2, P3 and P7) values were higher than the mean contents known in Spanish industrial, agricultural and natural soils (Roca-Perez et al., 2010), while Cr, Cu, Ni (except P1, P7 and P8) and Pb (except P1 and P5) were lower than industrial soils (Roca-Perez et al., 2010). As the study sites lie in a forest far from urban centres and industrial areas, our results seem to indicate that Ba mining activity is the main source of pollution by Cd, Co, Zn, Ni and Pb in our soils. It is noteworthy that the mean Cr, Cu, Ni, Pb and Zn values exceeded the maximum allowed values (63, 64, 50, 140 and 200 mg kg^−1^, respectively) for parkland soil use (CCME, 2007). Hence, these levels would indicate that the mine’s forest surroundings are unsuitable for outdoor activities. In general terms, the Cd, Co, Cu, Ni, Pb and Zn contents in most of the sampled soils were much higher than the generic reference levels legislated for the protection of ecosystems in Catalonia (BOE, 2009). This means that these soils are polluted.

The correlation coefficients among the physical, chemical properties and elements content in soil are shown in Table 4. SOM positively correlated with EC, CEC and Cr. This result was corroborated by other studies (Kabata-Pendias & Pendias, 2001; Roca-Perez et al., 2010; Rodríguez et al., 2006; Tume et al., 2006a, b) about the affinity of certain elements and SOM because of their high sorption capacity in relation to many pollutants, including heavy metals, which may result in their immobilisation and could, consequently, affect the protection of food and groundwater against pollution (Kwiatkowska-Malina, 2018; Roca-Perez et al., 2010). A positive significant correlation was obtained for Cd–Co, Cd–Ni, Cd–Pb; Pb–Ni, Pb–Co, Ni–Co and Cu–Fe. Thus, strong correlations between some elements reveal that their sources of pollution are the same (Gil et al., 2004; Hosseini et al., 2022). Some of these significant correlations are related by the occurrence of minor sulphide minerals in Ba veins, or are possibly due to adsorption by Fe-oxy-hydroxides (Alizadeh-Kouskuie et al., 2020, Tume et al., 2006a, b). The presence of minerals like chalcopyrite and asbolane at the studied mine could be related to the correlations between Cu–Fe and Co–Ni, respectively. In addition to the different degree of weathering and the proportion of minerals, other factors like topographical characteristics and physico-chemical soil properties can modify the relations between them.

**Table 4:** Pearson correlations coefficients (*n* = 8) among PHEs concentrations, clay, pH, EC, SOM and CEC

	Clay	pH	^a^EC	^b^SOM	^c^CEC	Cd	Co	Cr	Cu	Fe	Ni	Pb	Zn
Clay	1												
pH	0.718	1											
^a^EC	0.311	0.353	1										
^b^SOM	− 0.370	0.199	0.947**	1									
^c^CEC	− 0.677	− 0.083	0.873**	0.953**	1								
Cd	− 0.043	− 0.091	− 0.084	− 0.048	− 0.087	1							
Co	− 0.046	− 0.071	− 0.244	− 0.181	− 0.258	0.883**	1						
Cr	0.352	0.715*	0.824*	0.743*	0.557	− 0.314	− 0.355	1					
Cu	− 0.276	− 0.638	− 0.368	− 0.292	− 0.106	0.519	0.248	− 0.693	1				
Fe	− 0.462	− 0.721*	− 0.418	− 0.390	− 0.208	0.644	0.545	− 0.785*	0.762*	1			
Ni	0.062	− 0.309	− 0.513	− 0.394	− 0.361	0.796*	0.764*	− 0.705	0.651	0.659	1		
Pb	− 0.150	− 0.160	− 0.452	− 0.396	− 0.432	0.905**	0.927**	− 0.523	0.475	0.674	0.841**	1	
Zn	− 0.136	− 0.069	0.590	0.544	0.637	− 0.758*	− 0.807*	0.478	− 0.343	− 0.443	− 0.814*	− 0.923**	1

*p < 0.05, **p < 0.01

^a^Electrical conductivity

^b^Soil organic matter

^c^Cation exchange capacity

The Cd (at P1, P2 and P3), Cu (at P1, P7 and P8), Ni (at P1, P5, P6 and P8), Pb (at P1, P3–P8) and Zn (in P1, P4, P5, P6 and P8) concentrations were higher than the toxicity limit value for plants in the soils reported by Mendez and Maier, (2008). Therefore, the vegetation that grows in these areas could be labelled as phytotolerant for surviving on such soils polluted by high Cd, Cu, Ni, Pb and Zn contents.

### PHEs concentrations in plants

The Cd, Co, Cr, Cu, Fe, Ni, Pb and Zn concentrations in the roots and shoots of the studied plant species are shown in Table 5. The results revealed that the roots of *Thymus vulgaris* L. presented the highest Co, Pb and Zn concentrations, while Cd, Cr and Cu were higher in *Festuca ovina* L. Both species were found at the P5 sampling point, where concentrations in soil were also considerable, but not as high as P1. *Biscutella laevigata* L. obtained the highest Co, Cr, Cu, Ni, Pb and Zn values in shoots at P6. The fact that we did not find the highest concentrations in the species sampled at this point at P1 could be related to *Saponaria ocymoides* L. and *Cistus albidus* L. being unable to bioaccumulate these metals despite higher soil concentrations. *T.vulgaris*,* F. ovina* and *B. laevigata* presented greater accumulation capacity for these elements despite finding these same elements at lower concentrations in the soils at P5 and P6 than at P1. The maximum Cu, Pb, and Zn contents in the studied species were lower than the maximum values obtained for different species at the polymetallic Carolina mine in the Peruvian Andes (Bech et al., 2016). The maximum Cd and Pb concentrations in the leaves, stalks and roots of the corn plants growing in the soils collected near the Zimapán Hidalgo mining zone (Mexico) were lower than our results (Armienta et al., 2020).

**Table 5:** Metal contents (mg kg^−1^) in the plant samples around the abandoned barite Atrevida mine (Tarragona, NE Spain)

Plant		Site	Cd	Co	Cr	Cu	Fe	Ni	Pb	Zn
*Cistus albidus* L.	Shoot	P1	2.51 ± 0.45	4.34 ± 0.17	13.17 ± 0.46	20.20 ± 0.85	3135.81 ± 175.09	8.37 ± 0.64	21.28 ± 1.98	116.28 ± 2.63
	Root	P1	2.78 ± 0.40	1.88 ± 0.28	4.25 ± 0.16	7.07 ± 0.41	389.97 ± 14.10	0.47 ± 0.06	6.27 ± 0.03	39.34 ± 5.45
*Saponaria ocymoides* L	Shoot	P1	1.99 ± 0.27	6.49 ± 0.36	21.09 ± 0.02	29.28 ± 0.30	9004.36 ± 11.82	9.26 ± 1.04	46.29 ± 3.11	129.73 ± 8.09
	Root	P1	3.20 ± 0.29	1.55 ± 0.21	2.33 ± 0.25	19.74 ± 0.16	1398.32 ± 130.56	3.84 ± 0.40	66.70 ± 1.17	87.25 ± 2.83
*Umbilicus rupestris* (Salisb.) Dandy.	Shoot	P2	0.29 ± 0.02	1.68 ± 0.23	7.23 ± 1.23	4.16 ± 0.21	110.24 ± 15.10	1.62 ± 0.23	< 1	52.71 ± 3.13
	Root	P2	1.09 ± 0.15	2.72 ± 0.25	33.00 ± 3.05	16.02 ± 1.81	12,171.71 ± 398.40	7.89 ± 0.89	18.82 ± 0.10	109.53 ± 2.45
*Saxifraga fragilis* Schrank	Shoot	P2	< 0.1	1.68 ± 0.02	4.24 ± 0.48	3.94 ± 0.08	243.89 ± 39.44	0.91 ± 0.06	< 1	43.93 ± 2.55
	Root	P2	< 0.1	1.68 ± 0.01	12.66 ± 0.30	5.53 ± 0.17	832.87 ± 26.68	2.44 ± 0.30	< 1	72.73 ± 5.87
*Biscutella laevigata* L.	Shoot	P3	< 0.1	2.37 ± 0.23	11.05 ± 0.10	11.57 ± 0.97	787.23 ± 32.85	3.03 ± 0.07	2.65 ± 0.19	27.47 ± 4.81
	Root	P3	< 0.1	1.68 ± 0.09	1.46 ± 0.20	3.79 ± 0.13	412.99 ± 4.23	2.80 ± 0.14	< 1	14.94 ± 0.48
	Shoot	P5	0.34 ± 0.06	17.03 ± 0.11	4.76 ± 0.33	9.05 ± 0.21	3564.40 ± 109.45	17.17 ± 1.57	28.12 ± 0.32	158.03 ± 8.52
	Root	P5	0.14 ± 0.01	3.15 ± 0.04	1.07 ± 0.03	4.93 ± 0.08	897.25 ± 18.73	10.40 ± 0.59	10.00 ± 0.22	62.19 ± 3.02
	Shoot	P6	1.36 ± 0.23	39.34 ± 0.77	58.87 ± 8.81	29.22 ± 0.28	15,437.78 ± 354.65	30.85 ± 0.03	198.93 ± 0.77	310.09 ± 21.34
	Root	P6	0.22 ± 0.02	10.09 ± 0.21	13.76 ± 0.19	11.24 ± 0.31	1504.19 ± 21.47	15.87 ± 1.91	47.87 ± 2.94	116.09 ± 8.35
*Crepis pulchra* L.	Shoot	P3	1.09 ± 0.12	1.88 ± 0.27	4.24 ± 0.17	23.33 ± 2.38	^a^na	0.91 ± 0.06	< 1	39.71 ± 2.00
	Root	P3	1.54 ± 0.24	2.03 ± 0.05	3.06 ± 0.15	34.41 ± 0.28	1224.93 ± 100.30	3.35 ± 0.18	8.06 ± 0.16	82.41 ± 3.20
*Arrhenatherum elatiu*s (L.) P.Beauv. ex J.Presl & C.Presl	Shoot	P3	< 0.1	^a^na	2.93 ± 0.08	6.20 ± 0.28	^a^na	< 0.1	< 1	14.27 ± 2.56
	Root	P3	< 0.1	2.00 ± 0.07	5.41 ± 0.28	4.33 ± 0.23	274.35 ± 18.87	< 0.1	< 1	10.52 ± 1.39
*Dactylis glomerata* L.	Shoot	P3	< 0.1	1.68 ± 0.08	4.25 ± 0.18	7.40 ± 0.03	135.67 ± 19.33	0.90 ± 0.08	< 1	20.33 ± 2.31
	Root	P3	< 0.1	5.03 ± 0.03	2.94 ± 0.10	17.68 ± 0.74	^a^na	1.81 ± 0.08	3.12 ± 0.02	41.30 ± 5.89
*Lactuca viminea* (L.) J.Presl i C.Presl	Shoot	P4	2.44 ± 0.12	0.27 ± 0.04	1.37 ± 0.07	10.35 ± 0.48	704.33 ± 8.95	1.74 ± 0.08	1.98 ± 0.23	65.13 ± 2.77
	Root	P4	1.39 ± 0.20	1.35 ± 0.18	6.68 ± 0.46	5.66 ± 0.73	466.56 ± 20.58	0.98 ± 0.11	< 1	21.05 ± 3.39
*Silene italica* Pers. (L.)	Shoot	P4	< 0.1	3.02 ± 0.49	24.07 ± 0.16	8.11 ± 0.76	3391.37 ± 373.88	5.46 ± 0.02	15.90 ± 0.39	62.82 ± 5.09
	Root	P4	0.52 ± 0.01	1.54 ± 0.08	4.61 ± 0.13	7.29 ± 0.42	2981.32 ± 175.82	3.08 ± 0.14	34.70 ± 2.68	51.68 ± 2.28
*Thymus vulgaris* L.	Shoot	P5	< 0.1	3.13 ± 0.12	8.43 ± 0.27	7.68 ± 0.47	1165.22 ± 172.84	3.30 ± 0.30	10.96 ± 1.40	53.99 ± 3.41
	Root	P5	2.10 ± 0.08	50.76 ± 7.47	95.32 ± 5.71	54.01 ± 4.78	55,133.06 ± 185.35	50.20 ± 4.22	360.39 ± 23.71	379.33 ± 31.25
*Galium lucidum* All.	Shoot	P5	1.75 ± 0.14	1.90 ± 0.13	< 1	9.84 ± 0.46	275.49 ± 20.49	4.93 ± 0.80	9.34 ± 0.47	119.34 ± 4.41
	Root	P5	3.31 ± 0.21	7.91 ± 0.13	1.88 ± 0.11	20.22 ± 0.28	916.46 ± 31.76	22.71 ± 1.03	226.50 ± 27.58	152.24 ± 6.00
*Dactylis glomerata* L.	Shoot	P5	0.27 ± 0.05	1.25 ± 0.14	12.73 ± 1.02	6.29 ± 1.02	705.06 ± 22.71	4.53 ± 0.63	3.90 ± 0.42	43.81 ± 2.33
	Root	P5	1.02 ± 0.11	11.76 ± 1.02	25.85 ± 0.21	31.07 ± 1.41	55,659.57 ± 195.96	7.16 ± 0.19	66.75 ± 1.70	81.04 ± 0.65
	Shoot	P6	0.48 ± 0.03	3.45 ± 0.10	6.93 ± 0.93	8.81 ± 0.80	3350.74 ± 87.31	8.75 ± 0.19	18.29 ± 0.67	56.59 ± 1.21
	Root	P6	0.86 ± 0.03	8.23 ± 0.21	11.14 ± 0.18	19.43 ± 1.26	8410.64 ± 273.84	17.90 ± 1.92	38.48 ± 0.89	109.26 ± 9.87
*Euphorbia nicaeensis* All.	Shoot	P5	3.76 ± 0.36	2.30 ± 0.31	4.25 ± 0.18	14.53 ± 1.03	254.70 ± 19.37	4.27 ± 0.30	< 1	76.50 ± 0.00
	Root	P5	3.65 ± 0.21	4.23 ± 0.11	5.54 ± 0.10	28.19 ± 0.21	2598.81 ± 152.46	6.49 ± 0.33	18.18 ± 0.75	67.76 ± 3.69
*Festuca ovina* L.	Shoot	P5	0.22 ± 0.01	2.30 ± 0.32	8.46 ± 0.19	20.39 ± 1.77	686.84 ± 39.36	3.95 ± 0.44	7.83 ± 0.26	45.39 ± 1.41
	Root	P5	5.49 ± 0.29	43.26 ± 4.33	82.16 ± 3.20	57.36 ± 8.53	58,158.38 ± 221.15	44.51 ± 6.37	271.58 ± 9.31	310.69 ± 19.83
*Allium sphaerocephalon* L.	Shoot	P6	< 0.1	0.85 ± 0.04	1.47 ± 0.21	2.57 ± 0.16	38.87 ± 5.47	< 0.1	< 1	13.52 ± 0.55
	Root	P6	< 0.1	0.84 ± 0.06	1.44 ± 0.14	4.68 ± 0.38	131.10 ± 15.69	< 0.1	< 1	20.53 ± 1.15
*Antirrhinum majus* L.	Shoot	P6	< 0.1	0.55 ± 0.04	1.11 ± 0.13	9.25 ± 0.71	569.17 ± 25.70	< 0.1	< 1	34.44 ± 0.83
	Root	P6	< 0.1	1.82 ± 0.27	12.37 ± 0.16	8.84 ± 1.48	^a^na	4.62 ± 0.52	3.05 ± 0.21	52.94 ± 3.35
*Cirsium acaule* (L.) Scop	Shoot	P6	0.66 ± 0.04	1.65 ± 0.23	4.25 ± 0.33	12.05 ± 0.41	195.42 ± 18.98	0.91 ± 0.03	< 1	41.43 ± 3.79
	Root	P6	0.47 ± 0.03	2.08 ± 0.13	33.19 ± 0.88	14.85 ± 0.92	1845.24 ± 176.44	102.25 ± 4.59	14.83 ± 1.40	44.93 ± 5.52
*Petrorhagia prolifera* P.W.Ball & Heywood (L.)	Shoot	P6	< 0.1	< 0.1	^a^na	^a^na	^a^na	^a^na	^a^na	^a^na
	Root	P6	0.60 ± 0.03	2.05 ± 0.10	2.24 ± 0.03	5.37 ± 0.17	608.06 ± 33.86	3.82 ± 0.32	5.00 ± 0.78	36.66 ± 1.08
*Sedum rupestre* L.	Shoot	P7	< 0.1	< 0.1	1.62 ± 0.03	5.85 ± 0.52	471.39 ± 19.25	1.48 ± 0.15	1.25 ± 0.18	20.96 ± 2.10
	Root	P7	0.11 ± 0.01	0.22 ± 0.01	3.16 ± 0.17	7.59 ± 0.57	1110.12 ± 118.96	2.05 ± 0.08	3.60 ± 0.50	20.75 ± 1.28
*Geranium robertianum* L.	Shoot	P7	0.13 ± 0.01	0.10 ± 0.01	1.64 ± 0.06	9.92 ± 1.36	652.92 ± 15.44	2.13 ± 0.16	3.86 ± 0.42	34.79 ± 1.58
	Root	P7	0.85 ± 0.03	1.35 ± 0.15	9.14 ± 0.35	27.81 ± 1.86	3507.55 ± 14.28	6.90 ± 0.50	18.13 ± 0.45	84.39 ± 1.20
*Tanacetum corymbosum* (L.) Sch. Bip.	Shoot	P7	1.53 ± 0.11	< 0.1	< 1	14.77 ± 0.11	233.48 ± 12.59	3.33 ± 0.18	< 1	56.52 ± 1.85
	Root	P7	3.11 ± 0.12	2.40 ± 0.28	33.75 ± 1.71	46.82 ± 0.57	11,393.29 ± 429.51	10.39 ± 0.37	30.50 ± 1.35	102.36 ± 3.16
	Shoot	P8	1.76 ± 0.05	0.84 ± 0.08	8.45 ± 0.28	17.15 ± 0.71	440.16 ± 15.61	3.49 ± 0.31	1.60 ± 0.14	56.40 ± 4.38
	Root	P8	1.07 ± 0.10	3.81 ± 0.14	6.37 ± 0.30	34.40 ± 0.64	3935.38 ± 176.24	7.37 ± 0.11	7.45 ± 0.14	31.80 ± 3.77
*Asphodelus cerasiferus* Gay	Shoot	P8	1.21 ± 0.08	0.24 ± 0.04	5.66 ± 0.33	9.59 ± 0.23	1662.86 ± 45.05	5.88 ± 0.28	5.67 ± 0.25	33.89 ± 1.46
	Root	P8	< 0.1	2.30 ± 0.31	9.11 ± 0.66	19.12 ± 0.04	2393.56 ± 140.56	3.30 ± 0.28	3.9 ± 0.07	38.52 ± 2.11

Mean ± standard deviation; *n* = 3

^a^Not analysed

In the soils near the Mangampeta and Vemula Ba mines (India), Raghu (2001) found similar or slightly higher Cd, Co, Pb, Zn and Ni concentrations in plants, while Cr and Cu obtained lower and higher values, respectively, than those herein reported. In soils close to the ancient Pb/Ba mine Maria in Catalonia, NE Spain (Poschenrieder et al., 2012), the Cd and Zn contents in *Smilax aspera* and *Silene sclerocarpa* were similar or slightly lower, while Pb was higher than in the Atrevida mine plants.

The species belonging to the genus *Festuca* (i.e. *Festuca arvernensis*) display good tolerance to heavy metals in soil, but do not show accumulation in aerial parts (Escarré et al., 2011), while *Festuca ovina* is able to accumulate more Cu in root cells (Ebrahimi & Madrid, 2014), and Cu and Zn in the aboveground biomass (Gawryluk et al., 2020). In the soils polluted by Cd, Pb and Ni, *Thymus vugaris* accumulated these elements in roots, but not in aerial parts. Therefore, it is not a hyperaccumulating plant (Lydakis-Simantiris et al., 2016). *B. laevigata* is a metal hyperaccumulating species that grows in both polluted and non-polluted soils. Several studies highlight the bioaccumulative capacity of metals by this species in soils polluted by mining activity (Escarré et al., 2011; Muszyńska et al., 2017; Pavoni et al., 2017). Our results show that *B. laevigata* is better capable of accumulating heavy metals in aerial parts than the other species studied at P6. According to Kramer (2010), the critical toxicity level for Cd is 6–10 μg g^−1^, 0.4—several μg g^−1^for Co, 20–30 μg g^−1^ for Cu, 10–50 μg g^−1^for Ni, 0.6–28 μg g^−1^for Pb and 100–300 μg g^−1^for Zn in the aerial parts of different taxa. Thus, our results reveal that the Co, Cu, Ni, Pb and Zn contents in *B. laevigata* are on the limit or exceed the critical toxicity level set for these elements. However, the values obtained in the aerial parts of the studied species were lower than the concentrations indicated by Kabata-Pendias and Mukherjee (2007) and by Yoon et al. (2006), who considered that certain species were hyperaccumulators.

### The elements correlation between soil and plants

In order to evaluate the relations of elements in soil and plants, the correlation coefficients of the elements concentrations in soil, roots and shoots were determined (Table 6). The significant correlations found in the Co, Cu, Ni, Pb and Zn contents in soil and all the studied plants together indicated that the variation in the content of these elements in soil strongly influenced their contents in plants. Stefanowicz et al. (2016) indicated that wide variations in soil PHEs content leads to significant correlations between soil and plants. A positive correlation demonstrates that soil pollution would induce plant pollution (Ko et al., 2020).

**Table 6:** The Spearman correlation coefficients for Co, Cu, Ni, Pb and Zn in soil, roots and shoots

PHEs		Soil	Root	Shoot
Co (*n* = 23)	Soil	1		
	Root	0.578**	1	
	Shoot	0.490*	0.342	1
Cu (*n* = 26)	Soil	1		
	Root	0.432*	1	
	Shoot	0.392*	0.399*	1
Ni (*n* = 23)	Soil	1		
	Root	0.356	1	
	Shoot	0.633**	0.239	1
Pb (*n* = 22)	Soil	1		
	Root	0.454*	1	
	Shoot	0.145	0.262	1
Zn (*n* = 27)	Soil	1		
	Root	0.252	1	
	Shoot	0.506**	0.510**	1

*p < 0.05, **p < 0.01

### Accumulation and translocation of PHEs in plants

Plants’ accumulation and translocation potentials can be estimated by the BCF and BAF. Thus, these indexes reflect the capacity of plants to absorb and accumulate. If these indexes are ≤ 1, it implies that the plant can only absorb the element and does not have the capacity to bioconcentration or bioaccumulation for a specific element (Hosseini et al., 2022). In this study, the values of these ratios were below 1, except for the BCF in *U. rupestris* at site P2 for Cr (1.45), in *F. ovina* at site P5 for Cr (1.23) and in *T. corymbosum* at site P7 for Pb (1.18). Organic acids secreted by roots can modify metal solubility in soil insoluble forms, which can contribute to adsorption and absorption by the root system and, thus, their accumulation in roots (Ortiz-Oliveros et al., 2021). The BAF values were above 1 in *L. viminea* at site P4 for Cd (1.44) and in *B. laevigata* at site P6 for Pb (1.22). In line with what Bech et al. (2012a) reported, the few species with RAF (or BCF) and SAF (or BAF) values above 1 could be because the pseudo-total, and not the extractable soil metal concentration, was used herein for the RAF (or BCF) and SAF (or BAF) calculations. However, Buscaroli (2017) concluded that when studies are carried out to evaluate the bioaccumulation factors of metals in plants, the pseudo-total or available element fraction in soils seems more suitable. Reeves (2006) reported that the SAF (or BAF) is not a good measure of plants’ capacity to accumulate metals. Several factors can affect element uptake by plants, such as soil pH, CEC, plant variety and plant growth stages (Buscaroli, 2017). Although the studied soils have a slightly acid pH and loam and clay loam textures, the high SOM content in these soils would favour PHEs retention processes in soil as opposed to absorption by plants. This could be related to the low accumulation observed in the studied plants. In fact, the high sorption capacity of heavy metals by organic matter can lead to their immobilisation in soil and would, therefore, not favour bioaccumulation in plants (Kwiatkowska-Malina, 2018).

The TF allows the degree of transfer and the redistribution of heavy metals between roots and shoots to be known. Plants with strong transferability that grow in soil with high levels of heavy metals can transfer these elements from the rhizhosphere to aerial parts by protecting roots from toxicity (Wu et al., 2021). Plant species with a TF over 1 are suitable for phytoextraction. This index shows that plants translocate heavy metals most efficiently to aerial parts (Bech et al., 2012a, 2012b, 2016; Hossain et al., 2021; Hosseini et al., 2022; Tavili et al., 2021). In this study, the TF values ranged between 0.2 and 18.0 for PHEs (Table 7 presents the species with a TF > 1). High TF values were obtained in *C. albidus* at P1 for both Fe (TF = 8.04) and Ni (TF = 17.81), and also in *S. ocymoides* for Cr (TF = 9.05), and in *B. laevigata* at P6 site for Fe (TF = 8.04), which displayed good translocating ability for these elements in the above plant species.

**Table 7:** The translocation factor, TF^(a)^, of the natural plants from the barite Atrevida mine. Only those species with more than one element with a TF value over 1 are shown

Species	Soil site	Cd	Co	Cr	Cu	Fe	Ni	Pb	Zn
*Cistus albidus*	P1	0.90	2.31	3.10	2.86	8.04	17.81	3.39	2.96
*Saponaria ocymoides*	P1	0.62	4.19	9.05	1.48	6.44	2.41	0.69	1.49
*Biscutella laevigata*	P3	–	1.41	7.57	3.05	1.92	1.08	–	1.84
*Biscutella laevigata*	P5	2.43	5.41	4.45	1.83	3.97	1.65	2.81	2.54
*Biscutella laevigata*	P6	6.18	3.89	4.28	2.60	10.26	1.94	4.15	2.67
*Lactuca viminea*	P4	1.76	0.20	0.21	1.83	1.51	1.78	–	3.09
*Silene italica*	P4	–	1.96	5.22	1.11	1.14	1.77	0.46	1.21

^a^TF: Metal concentration ratio of plant shoot to root– not determined

In general, BCF and BAF values were lower than 1 in most of the plants and PHEs studied, however, we found a greater number of elements, for same species, with TF values higher than 1. Thus, these species could transfer PHEs from the roots to the aerial part as a strategy to protect the root system from the toxicity caused by the high concentration of these elements (Wu et al., 2021, 2023).

It is noteworthy that *B. laevigata* had a TF above 1 for most of the elements analysed at the P3, P5 and P6 where this species was found, which is an indicator of good translocation capacity to plant aerial parts for the analysed elements. So according to the TF criterion, this species can be considered a hyperaccumulator of Cd, Co, Cr, Cu, Fe, Ni, Pb and Zn because they had a TF value above 1. Pavoni et al. (2017) report similar results for *B. laevigata* from a *Z*n–Pb mine in the NE Italian Alps. Although the SAF (or BAF) values were not above 1 in the studied elements, except Pb, *B. laevigata* can be considered a hyperaccumulator of Co, Cu, Ni, Pb and Zn according to the following criteria: (a) the concentrations of these elements equalled or exceeded the values, which indicates critical toxicity for plants (Kramer, 2010); (b) their TF values were higher than 1 (Bech et al., 2012a, 2012b, 2016).

## Conclusions

The present study examines the current pollution status of some potentially harmful elements in the soil and vegetation in the vicinity of the Atrevida mine, NE Spain. The Cd, Co, Cr, Cu, Ni and Pb concentrations at most sampling sites are higher than the median values for agricultural soil in S Europe and the generic reference levels for Catalonia. The source of these elements is attributed mainly to mining activities associated with Ba extraction in the nineteenth and mid-twentieth centuries. Marked heterogeneity appears in the studied soils around the mining areas with the most soil pollution.

The highest PHEs concentrations in roots and shoots are, respectively, found in *Thymus vulgaris* and *Festuca ovina* at P5, and in *Biscutella laevigata* at P6. No high values are noted in the species growing in the most polluted soil (*Saponaria ocymoides* and *Cistus albidus*). These species can tolerate high PHEs concentrations, but without bioaccumulation capacity. The BCF and BAF indices generally show that these elements do not tend to accumulate in the studied plant species. These indices are not directly related to plants’ ability to tolerate the elements in question, but might be attributed to high loads in these soils. However, both the BCF values for *F. ovina* at P5 for Cr and the BAF values for *B. laevigate* at P6 exceed 1. The TF values for *B. laevigata* are higher in most of the studied PHEs regardless of sampling site. Thus, we suggest that *B. laevigata* is a PHEs accumulator given its ability to accumulate high Cd, Co, Cr, Cu, Fe, Ni, Pb and Zn contents in shoots in polluted soils, and is well able to transport these metals from roots to shoots.

## Data Availability

The datasets used and/or analysed during the current study are available from the corresponding author on reasonable request.

## Declarations

### Animal research

No animals were involved in the study.
